# Estimation of body weight using anthropometric parameters in Sri Lankan hospitalized adult patients

**DOI:** 10.1371/journal.pone.0290895

**Published:** 2023-09-01

**Authors:** H. M. M. T. B. Herath, K. W. S. M. Wijayawardhana, U. I. Wickramarachchi, Sunethra Senanayake, Bimsara Senanayake, Chaturaka Rodrigo

**Affiliations:** 1 Senior Registrar in Neurology, National Hospital of Sri Lanka, Colombo, Sri Lanka; 2 Faculty of Medicine, University of Colombo Sri Lanka, Colombo, Sri Lanka; 3 Faculty of Medicine, University of Moratuwa, Sri Lanka, Moratuwa, Sri Lanka; 4 Neurology Department, Consultant Neurologist, National Hospital of Sri Lanka, Sri Lanka; 5 Department of Pathology, School of Medical Sciences, UNSW Sydney, Sydney, NSW, Australia; Universitair Kinderziekenhuis Koningin Fabiola: Hopital Universitaire des Enfants Reine Fabiola, BELGIUM

## Abstract

Body weight is an important clinical parameter for accurate dosing of drugs with a narrow therapeutic window, However, it is difficult to measure the body weight of a patient if they cannot stand on a scale. There are several anthropometrics-based equations to estimate the body weight, but most of these are derived from white Caucasian populations and are not validated for South Asians. This study aimed to validate existing anthropometrics-based weight estimation equations and develop a new equation for the same purpose for Sri Lankan adults. This prospective study was conducted at the National Hospital of Sri Lanka over a 6-month period, split into a development and a validation phase. During the development phase, estimated body weight of patients by doctors and nurses and patients themselves were noted and compared against their actual body weight. In addition, 13 anthropometric measurements were taken, which were used to validate 12 anthropometrics-based equations to estimate body weight described in literature previously. Two new gender specific regression models to estimate the body weight in the local population was also derived and validated. A total of 502 (males = 249) and 217 (males = 108) patients were recruited for the development and validation phases respectively. Both doctors and patients had comparable accuracy in predicting body weight (p>0.05). All anthropometric based equations were significantly correlated with actual body weight (correlation coefficients: 0.741–0.869), and the new equations derived from the local data performed similarly to the best performing equation identified from the literature during validation phase. However, even the best of these equations could not outperform patient/physician estimates. When the patient weight cannot be measured, an estimate by the patient or the doctor may be the best substitute.

## Introduction

The dose of several medications used in the emergencies including that for thrombolysis in stroke depends on the patients’ actual body weight (ABW) [1]. Body weight has implications for critically ill patients’ drug including vasopressors, sedative drugs, induction agents, anticoagulations, antibiotics, analgesic drugs and intravenous fluids, fluid regimens in burn patients. Many of these drugs used do require more precise dosing. Administering a larger than required dose of recombinant tissue plasminogen activator for thrombolysis in acute ischaemic stroke increases the risk of fatal intracerebral haemorrhage, while a lower than required dose may be ineffective. However, measuring the patient’s ABW is difficult in an emergency setting because some are unable to stand on a scale without assistance (e.g., patients with myocardial infarctions, stroke, severe burns and sepsis), and bedbound scales are not available in many centres in middle- and low- income countries. In such situations doctors and nurses estimate the body weight of the patient. The accuracy of these “guessed” estimates are subjective, cannot be reproduced, and depends on clinical exposure and expertise of the healthcare worker [2–9]. According to previous literature. healthcare workers were more likely to underestimate weight of females (compared to males) [6]and be less accurate in estimates for underweight and obese patients [7]. The alternative is to ask the patient what their weight is, but not all patients frequently measure themselves, and some may be incapacitated within the critical time window when treatment decisions must be made fast. As an alternative, calculating an estimate of body weight based on other anthropometric measurements, at least in theory, is a more objective and reproducible method.

There are several different equations and nomograms available in the literature for this purpose which are summarised in S1 Table. All these of these collectively use 13 anthropometric measurements which can be observed in a supine patient. The definitions of these anthropometric measurements are explained in S2 Table. However, almost all these equations were derived from white Caucasian populations (United states of America– 5, United Kingdom– 2, Germany, Italy, and Latvia– 1 each). The remaining were from Mexico, Brazil and Hong Kong, and none are representative of the South Asian adults. There are physical differences between Asians and Caucasians [10] which may affect the accuracy of anthropometric measurements. For example Asians have more subcutaneous fat than their Caucasian counterparts which results in a larger mid arm circumference (MAC) and skinfold thickness readings in general [11]. Therefore, the existing nomograms and equations must be validated for South Asian patients [12].

Sri Lanka is a South Asian country with an estimated population size of 22 million people. It is a low-middle income country with a per capita annual income of USD 3815 in 2021. Its per capita health expenditure was US Dollars 157 in 2018 which was 3.9% of Gross domestic product [13]. As clinicians on the ground, we do not have access to bedbound scales in most centres and hospitals. Thus, to avoid overprescribing or under prescribing lifesaving medication, it is essential to have an objective way of predicting actual body weight of patients who cannot stand up on the scale to be measured. The aims of this prospective study were to a) to assess the accuracy of weight estimations by healthcare workers and patients, b) to validate anthropometric measurement-based weight estimation equations in prior literature for Sri Lankan adults, and c) to develop and validate a new body weight estimation equation for Sri Lankan adults.

## Materials and methods

This was a prospective, observational study conducted in two (development and validation) phases. Ambulatory consenting patients admitted to two neurology wards in the National Hospital of Sri Lanka (in Colombo), who were 18 years of age or older, clinically stable, and able to stand on a weight scale without assistance were recruited. Patients with an altered mental status, those with limb amputations or chronic diseases likely to cause fluid retention (e.g., liver cirrhosis, acute or chronic kidney disease, Cushing syndrome, symptomatic heart failure), were excluded. Simple obesity was not an exclusion criterion.

In the development cohort, eligible patients were recruited by consecutive sampling (from April 2022 to August 2022). Patients as well as attending doctors and nurses were asked to estimate the weight of the patient as a “best guess”. All healthcare workers in these wards are also involved in thrombolysis of stroke patients are hence have prior experience in prescribing medications according to estimated body weights. The doctors and nurses had at least 1 year of experience in practice, but there was no stratification of healthcare workers according to their experience. Instead, two different measurements from members of the same category were taken and the mean value was entered per patient. Due to rostering, it was not possible to get the same set of healthcare workers to assess all patients. All patients and health care staff were blinded to the patient’s ABW when making estimates. Next, the investigators took anthropometric measurements given in S2 Table for all patients. The circumferences were measured by a tape as the average of two measurements and were rounded up to the nearest centimetre. The skin fold thicknesses were measured by Bozeera skinfold caliper Pro (China) as an average of two measurements and rounded up to the nearest millimetre. The estimated body weight was calculated from the anthropometric measurements for all patients, using all equations listed in S1 Table. Finally, the patients were asked to stand on a digital weighing scale to measure their body weight (mean of two measurements). Patients were weighed wearing the clothes (gross weight) and we pre-weighted a set of cloths an average patient is likely to wear in the hospital ward and subtracted this value from gross weight. For circumference measures the clothing were adjusted to expose the relevant body area prior to measurement. All patients were barefoot and all accessories such as jewellery, keys, wallets, and cell phones were removed before weighing. No demographic data were collected apart from age and gender. Name and address were collected to avoid duplicate entries, but these items were removed from the final dataset (de-identified) prior to analysis.

In the validation cohort, the same process was repeated, in the same hospital wards with same healthcare workers but in a different recruitment period (August 2022 –September 2022). The weight estimations from the best performing anthropometric-based equation identified in the previous phase were compared against a new gender specific equation (see below) derived from the data of the same phase for validation. This study was approved by the ethics review committee of the National Hospital of Sri Lanka (AAJ/ETH/COM/2021/November). Informed written consent was obtained from all participants prior to enrolment.

In the absence of a precedence in Sri Lanka to calculate sample sizes and given that most equations that were to be validated were derived by linear regression with up to 4 independent variables, Green’s rule of thumb (n = 50+8*number of predictors) was used to calculate the minimum sample size of the development cohort (n = 82) [14]. However, we also considered the median sample size (n = 231) of previous studies of similar design listed in S1 Table and decided to match this number per gender. For validation cohort sample size estimation was done according to the Green’s rule of thumb to test the coefficients of a linear regression equation (n = 104+ number of predictors) [14]. Data were analysed with Statistical Package of Social Science (SPSS, v25, IBM, USA). Descriptive statistics were summarised as measures of central tendency (mean or median) and dispersion (standard deviation or inter-quartile range) according to normality of distributions. The percentage errors of estimation in weight were calculated separately for patients, doctors and nurses. The observed percentage errors were converted to dichotomous variables based on 5% or 10% error cut-offs and compared between categories using the z-test for proportions. The ABW was correlated with predictions by different anthropometrics-based equations with spearman correlation. To derive a better fitting regression equation for body weight estimation with development phase data, all anthropometric measurements were first correlated with ABW to identify those with a correlation coefficient >0.3. These were cross-correlations against each other to define clusters of measurements where within cluster correlation coefficient >0.7, but the between cluster correlation coefficient was <0.7. One measure from each cluster were selected (easiest to measure, to avoid co-linearity) and a linear regression was done to derive a regression equation for prediction of ABW. These regression equations were tested in the validation cohort against the best performing anthropometrics-based equation identified from the literature. Modified Bland Altman plots were used to compare the weight estimations by patients and the best performing equations.

## Results

### Development cohort

A total of 502 patients were recruited for this cohort (males = 249, 49.6%, mean age 50.2 ± 26.8 years). Their mean ABW was 59.5 ± 12.4 kg (range: 31–116 kg). Body weight was estimated by patients on 491 (97.8%) instances and by healthcare workers for all patients. The instances where estimated body weight by each category had a >5% and > 10% error are summarized in Table 1. At both these cut-off levels, patients were as accurate as nurses or doctors while nurses had a significantly higher error rate compared to doctors (p<0.05). In the same table we calculated and compared instances with >20% error across categories as an extreme case scenario and found doctors are significantly less likely than nurses or patients to make such large errors in estimations (p<0.05).

**Table 1:** Percentage error in estimated body weight in each of the participant categories (development cohort).

Categories compared	Numbers	Percentages	P value*
*Error ≥ 5% of actual body weight*			
Patients vs. Doctors	209/491 vs. 208/502	42.6 vs. 41.4	0.719
Patients vs. Nurses	209/491 vs. 241/502	42.6 vs. 48.0	0.085
Doctors vs. Nurses	208/502 vs. 241/502	41.4 vs. 48.0	0.037
*Error ≥ 10% of actual body weight*			
Patients vs. Doctors	94/491 vs. 77/502	19.1 vs. 15.3	0.112
Patients vs. Nurses	94/491 vs. 112/502	19.1 vs. 22.3	0.219
Doctors vs. Nurses	77/502 vs. 112/502	15.3 vs. 22.3	0.005
*Error ≥ 20% of actual body weight*			
Patients vs. Doctors	23/491 vs. 2/502	4.7 vs. 0.4	<0.001
Patients vs. Nurses	23/491 vs. 26/502	4.7 vs. 5.2	0.719
Doctors vs. Nurses	2/502 vs. 26/502	0.4 vs. 5.2	<0.001

*Calculated with z test for proportions.

A summary of all anthropometric measurements of the derivation cohort is shown in S3 Table. The estimated weight was calculated for all patients using the 12 equations listed in S1 Table (nomogram from row 2 was excluded), and the results were correlated with the actual body weight (Table 2, S4 Table). Overall, the equation by Buckley et al. (row 1, S1 Table) had the best correlation with the actual body weight (spearman’s ρ: 0.869. p<0.001) [1]. Some studies derived their equations for specific demographic subgroups (e.g., equation by Bernal-Orozco et al. [12] was for females only), and these groups were separately considered in a sensitivity analysis. However, on all occasions the agreement with the ABW was better for the whole sample than with demographic subgroups.

**Table 2:** Spearman correlation coefficients between actual body weight and the estimated body weight calculated from each of the equations listed in S1 Table***.

Equation	Spearman correlation coefficient (p value)		
	For whole cohort (n = 502)	For people above 60 years* (n = 152)	For people above 65 years* (n = 100)
Buckley et al. [1]	0.869 (<0.001)		
Chumlea et al. [15]	0.830 (<0.001)		0.677 (<0.001)
Chumlea et al. [15]	0.828 (<0.001)		0.709 (<0.001)
Chumlea et al. [15]	0.784 (<0.001)		0.632 (<0.001)
Bernal–Orozco et al. [12]	0.762 (<0.001)**		
Balode et al. [16]	0.854 (<0.001)		0.808 (<0.001)
Donini et al. [17]	0.770 (<0.001)		0.616 (<0.001)
Lin et al. [18]	0.801 (<0.001)		
Atiea et al. [19]	0.741 (<0.001)		0.607 (<0.001)
Cattermole et al. [20]	0.770 (<0.001)		
Jung et al. [21]	0.744 (<0.001)	0.668 (<0.001)	
Rabito et al. [22]	0.848 (<0.001)		

*These equations were derived for these subgroups only

**For females only (n = 253)

*** Of studies presented in S1 Table, Lorenz et al only had a nomogram and Chittawatanarat et al. had multiple equations each considering two anthropometric measurements only–these are excluded in this table.

In the development cohort there was a statistically significant difference in ABW between males and females (61.2 vs. 57.9, p = 0.003, independent T test). Hence, two regression models were developed for males and females. For each gender, all anthropometric measurements and age were cross correlated against the ABW to select those with a Spearman correlation coefficient > 0.3. In this regard the data points could be described by a linear relationship just as well as by more complex patterns of relationships (S5 Table). Age and knee height for males and age, knee height and tibial length for females did not meet this criterion. The Spearman correlation coefficient was then compared across remaining measurements to identify those highly correlated with each other (correlation coefficient > 0.7), and from such clusters one representative variable was selected to avoid co-linearity. On this basis mid-arm circumference (MAC) and abdominal circumference (AC), tibial length (TL) and triceps skin-fold thickness (TST) were selected for males as independent variables. Mid-arm circumference (MAC), neck circumference (NC) and chest circumference (CC), subscapular skin-fold thickness (SST), waist skinfold thickness (WST) and triceps skin fold thickness (TST)were selected for females. The linear regression model for males had a R^2^ value of 0.824 (82.4% of variation was explained by the independent variables) and all input variables were independently associated with ABW (p<0.001, S6A Table). The regression model for females had a R^2^ value of 0.747 and only MAC, CC and NC were independently associated with ABW (p<0.001, S6B Table). Assumption testing (model diagnosis) for both regressions is presented in S7 Table. The derived regression equations (henceforth referred to as “new equations”) were as follows.


$$ABW_{male}=‐38.21+1.29*MAC+0.49*AC+0.565*TL+0.391*TST$$

$$ABW_{female}=‐20.128+0.968*MAC+0.273*NC+0.407*CC$$

### Validation cohort

A total of 217 patients were recruited for the validation cohort (males = 108, 49.8%, mean age 45.1 ± 15.7 years). Their mean ABW was 59.5 ± 12.9 kg (range: 31.6–112 kg). The comparisons between development and validation cohorts are shown in S8 Table. For males, there were no statistically significant differences for age or any of the anthropometric measurements of interest. For females, there were statistically significant differences in age and neck circumference. The actual body weight correlated statistically significantly with the predicted body weight from the new equation for both males and females (p<0.001). However, when compared to the patient estimated body weight, the new equation had significantly higher number of instances where the error was ≥ 5% (99/217 vs. 129/217, 45.6% vs. 59.4%, p = 0.004). However, the instances where the error was ≥ 10% (48/217 vs. 65/217, 22.1% vs 30%, p = 0.063) or ≥ 20% (17/217 vs. 9/217, 7.8% vs 4.1%, p = 0.105) were comparable.

When the weight estimations from the new equation was compared against the same by Buckley et al. (best performing regression model from S1 Table), for males, the new equation was slightly better (Spearman’s ρ: 0.851 vs. 0.821). The reverse was true for females (Spearman’s ρ: 0.923 vs. 0.935). The proportion of instances where the estimate by either equation had a ≥5%, ≥10% or ≥20% error from the ABW were also similar indicating equal performance (p>0.05, z-test for proportions).

The modified Bland Altman plots demonstrating the relationship of ABW vs. the patient estimated weight for males and females are shown in S1A and S1B Fig respectively. Similarly, the relationship between ABW vs. the estimated value from the new equation for males and females are shown in S1C and S1D Fig. Inspection of these plots suggest that in general, the new equation tends to overestimate the weight. for males, the equation is more accurate for the 45 – 80kg age range, whereas for females the range is even narrower between 40-60kg. In females, the error in overestimation increases when the ABW increases over 60kg.

## Discussion

This prospective study explored the validity of anthropometrics-based body weight estimation equations from literature which were derived from non-South Asian populations, in a Sri Lankan adult population. It also designed a new equation for body weight prediction using local population data which performed on par with the best fitting equation from the previous studies. However, the self-reported weight by patients or those guessed by doctors, outperformed those predicted by anthropometrics-based equations.

Several previous studies have compared the estimates by healthcare workers of patient body weight against the patient’s own estimate, and have concluded that patient’s estimate to be one the most reliable, if not the most reliable, out of all estimates assessed [1–3]. Even though the patient “guesses” his body weight for the study, it is likely this estimate is pre-informed by a weight measurement in the past (though it may not be recent). This may help the prediction to be more accurate than that of a healthcare worker who had a brief encounter with the patient. However, a healthcare worker who frequently estimates body weights of patients and then validates these estimations against the actual body weight (if measured later), may also be able to accurately predict it with experience. In this study the doctors working in neurology units who must frequently estimate the body weights of unconscious patients, also had an accuracy comparable to patient estimates. However, these findings cannot be generalised for all healthcare workers. Though patient estimates for body weight are favoured by current literature, there are instances where patients have misconceptions about their own weight specially if they are over- or underweight [23]. Similarly, when the patient is unable to talk or unconscious, a more objective method estimate body weight is needed which is immune from physician inexperience.

There have been many attempts to fill this void with anthropometrics-based weight estimation equations as shown in S1 Table. Most of these methods are based on multiple anthropometric measurements [1,15] while some are based on a single measurement [20]. However, some of these methods are only applicable to demographic subgroups (by gender or age) or within a range of body weight, and beyond these confines their accuracy falters. Furthermore, these equations are developed and validated in specific ethnic populations, and they cannot be directly applied to other ethnic groups without validation as they may have significantly different genetic and environmental influences on body build. All the equations (regression models) from previous literature tested on this Sri Lankan dataset had statistically significant correlations with the ABW though the correlation coefficients varied from 0.741 to 0.869. The new equations derived by us had correlation coefficients that matched the best of these previous equations [1]. Interestingly some of these equations from literature were only meant to be used in elderly people (>60 or 65 years), but they performed less well for these subgroups when compared to the whole sample (Table 1). Thus, it could be argued that the ethnic differences in body build may be greater in elderly, and weight estimation equations derived elsewhere should not be used in this subgroup without validation in the local population.

Despite the overall good correlation observed between the ABW and the estimated body weight by the new equations, the modified Bland Altman plots show the validity of the equation-based estimates to be less at higher ABWs, especially for females. In addition, throughout the entire range of ABW, the new equations tend to overestimate the weight. This was not seen with patient / doctor estimates where over- and underestimates were equally distributed. Thus, patient or doctor’s estimate of body weight may be superior to any of the anthropometrics-based equations, but when the patient is unconscious or unable to speak, we propose that the new equations derived here are a good alternative for weight estimation in Sri Lankan adult males with a ABW of 45–80 kg range, and for adult females with a ABW range of 40–60 kg. To our knowledge, there are no similar studies for Sri Lankan adults in literature though a height based weight estimation had been proposed for Sri Lankan children [24]. A post-mortem study that correlated head circumference with body weight of Sri Lankan adults found a statistically significant correlation for both genders, but the correlation coefficient for females was much lower compared to males (0.745 vs. 0.365). This study had a smaller sample size of only 156 cadavers, the body weight could have been influenced by post-mortem changes, and the findings were not validated in a living cohort [25].

As for limitations, this was a single centre study, and we did not record racial / ethnic identities of patients. However, the Colombo district where the National Hospital of Sri Lanka is located is the most populous district of Sri Lanka where more than 10% of the total population of the country lives (and more migrate daily for work). Hence, we assume the people seeking medical care at this hospital to be representative of the rest of the country. Sri Lanka is a small country with an area of approximately 65,000sqkm and its people are largely homogenous in body build despite various ethnic identities. Study was conducted in neurology wards, but the results can be used for other patients in emergency care and intensive care units as well. The healthcare workers who estimated weight of patients were not similar across patients, and this would have created an inter-observer bias. However, given that both development and validation phases were carried out within a six-month period, and that there were no major changes to the staff during this period, we believe these biases to be equally distributed across both phases of the study. Although anthropometrics-based regression equations may be best suited for individuals who are unable to report their own weight, we did not include any patients who are unconscious and unable to self-report their weight.

In conclusion, estimates by the patient or the attending doctor may be the most accurate and convenient estimate in instances where the actual body weight of the patient cannot be measured. However, if the patient is unconscious or cannot speak, two new gender specific anthropometrics-based regression equations presented here are likely to produce a reasonable estimate (±10kg of actual body weight) for Sri Lankan adult males between 45 – 80kg of weight and females between 40–60 kgs of weight.

## Supporting information

S1 Checklist Strobe statement check list of items included in the observational study. (DOCX)

S1 Fig 1A. Modified Bland-Altman plot of actual body weight vs. the difference between actual weight and the patient estimate (cohort: validation, gender: male). X axis- actual body weight (kg), Y axis–difference between actual body weight and patient estimate in kg. A positive value on y-axis indicates an underestimate by the patient and vice-versa. 1B. Modified Bland-Altman plot of actual body weight vs. the difference between actual body weight and the patient estimate (cohort: validation, gender: female). X axis- actual body weight (kg), Y axis–difference between actual body weight and patient estimate in kg. A positive value on y-axis indicates an underestimate by the patient and vice-versa. 1C. Modified Bland-Altman plot of actual body weight vs. the difference between actual weight and new equation estimate (cohort: validation, gender: male). X axis- actual body weight (kg), Y axis–difference between actual body weight and the equation estimate in kg. A positive value on y-axis indicates an underestimate by the equation and vice-versa. The observations are almost equally distributed on either side of the line of no difference (0.00 on y axis) between the 45 – 80kg range of ABW. 1D. Modified Bland-Altman plot of actual body weight vs. the difference between actual body weight and the patient estimate (cohort: validation, gender: female). X axis- actual body weight (kg), Y axis–difference between actual body weight and the equation estimate in kg. A positive value on y-axis indicates an underestimate by the equation and vice-versa. The equation generally overestimates body weight in females and this error keeps increasing after the ABW exceeds 60kg.
(TIFF)

S1 Table Anthropometrics-based regression models and nomograms to predict body weight from previous literature. (DOCX)

S2 Table Definitions of anthropometric measurements. (DOCX)

S3 Table Anthropometric measurements of all patients in the derivation cohort (n = 502). (DOCX)

S4 Table Percentage errors between actual body weight and the estimated body weight calculated from each of the equations listed in S1 Table. (DOCX)

S5 Table The correlation of each anthropometric measurement with actual body weight according to different patterns of distributions. (DOCX)

S6 Table a. Prediction of body weight with selected anthropometric measurements–summary of linear regression for males. b. Prediction of body weight with selected anthropometric measurements–summary of linear regression for females.
(DOCX)

S7 Table Model diagnosis of linear regression presented in S6 Table. (DOCX)

S8 Table Differences between validation and derivation cohorts for age, actual body weight and anthropometric measurements of interest that were selected for the regression equation. (DOCX)

## Abbreviations

ABW: actual body weight
MAC: mid-arm circumference
AC: abdominal circumference
TL: tibial length
TST: triceps skin-fold thickness
NC: neck circumference
CC: chest circumference
SST: subscapular skin-fold thickness
WST: waist skinfold thickness
