# Supplementary material for: Estimation of body weight using anthropometric parameters in Sri Lankan hospitalized adult patients
Source: PLoS One. 2023 Sep 1;18(9):e0290895. doi: 10.1371/journal.pone.0290895 (PMC10473512; doi:10.1371/journal.pone.0290895)
Supplement: S1 Table — (DOCX) [file pone.0290895.s003.docx]

Supplementary Table 1. Anthropometrics-based regression models and nomograms to predict body weight from previous literature.

| Study | Year | Country | Number of participants | Mean Age in years (SD or range) | Equation/nomogram | | Notes |
| --- | --- | --- | --- | --- | --- | --- | --- |
|  |  |  |  |  | Male | Female |  |
| Buckely et al. (1) | 2012 | United States | 307 | 41 (18–93) | -47.8 + 0.78 x (AC) + 1.06 x (TC) | -40.2 + 0.47 x (AC) + 1.30 x (TC) |  |
| Lorenz MW et al(2) | 2007 | Germany | 6962 | 19–90 | Nomogram | Nomogram |  |
| Chumlea et al. (3) | 1988 | United States | 228 | 65 - 104 | (MAC x 2.31) + (CC x 1.50)-50.10 | (MAC x 1.63) + (CC x 1.43)-37.46 |  |
| Chumlea et al. (3) | 1988 | United States | 228 | 65 - 104 | (MAC x 2.31) + (CC x 1.50)-50.10 | (MAC x 0.92) + (CC x 1.50) + (SST x 0.42)-26.19 |  |
| Chumlea et al. (3) | 1988 | United States | 228 | 65 - 104 | (MAC x 1.73) +(CC x 0.98) +(SST x 0.37) +(KH x 1.16)-81.69 | (MAC x 0.98) + (CC x 1.27) + (SST x 0.40) + (KH x 0.87)-62.35 |  |
| Bernal – Orozco et al. (4) | 2010 | Mexico | 43 | 84.3 ± 7.3 |  | (1.599 x KH) + (1.135 x MAC) + (0.735 x CC) + (0.621 x TST)–83.123 | The equation significantly overestimated weight by an average of 1.9 kg |
| Balode et al. (5) | 2015 | Latvia | 223 | ≥65 years | 0.709x AC +1.425 x MAC +1.083 x CC-68.968 | 0.853 x HC + 1.405x MAC+0.499x CC–79.355 |  |
| Donini et al. (6) | 1998 | Italy | 285 | 72.8 +/- 8 years old | 36.2 ( ln MAC)* + 42.47 (ln CC)* + 6.91 (ln SST)* + 0.8 (KH) - 253.7 | 1.41 (MAC) + 1.11 (CC) + 0.47 (SST) + 1.0 (KH) - 67.37 |  |
| Lin et al. (7) | 2009 | USA | 235 | 18-97 | KH x 1.10+MAC x 3.07-75.81 | KH x 1.10+MACx 2.81-66.04 |  |
| Atiea et al. (8) | 1994 | UK | 211 | 77.2 (63 - 99) in males and 78.1 (66 – 90), in females | -45.293 + 1.732x MAC + 0.687x CHC | 12.442 + 0.757 x TC + 0.484 x WST | Not as accurate for females compared to males |
| Cattermole et al. (9) | 2017 | UK | 6049 | 16-79 | 3.8484 × MAC −46.8585 | 3.8484 × MAC −46.8585 |  |
| Jung et al. (10) | 2004 | Hong Kong | 300 | 60-109 | [KH x 0.928 + MAC x 2.508 – Age x 0.144] - 42.543 | [ KH x 0.826 + MAC x 2.116 – Age x 0.133] – 31.486 |  |
| Rabito et al. (11) | 2006 | Brazil | 368 | 49 (SD=17) | 0.5759 x MAC + 0.5263 x AC + 1.2452 x CC - 4.8689 -32.9241 | 0.5759 x MAC + 0.5263 x AC + 1.2452 x CC - 4.8689 x 2 -32.9241 |  |
| Chittawatanarat et al. | 2012 | Thailand | 2000 |  | b1(covariate) + b2(height) + a* |  |  |

MAC = mid arm circumference, NC = neck circumference, CC = chest circumference, AC = abdominal circumference, WC= waist circumference, HC = hip circumference, TC = thigh circumference, CFC = calf circumference, KH = knee height, TL = tibial length, TST = triceps skinfold thickness, SST = subscapular skinfold thickness, WST = waist skinfold thickness, In = natural logarithm, ,

All measurements are taken in centimetres except in * where it is taken in inches

*covariates=Neck circumference, AC, CC, WC, HC, TC, CFC, AC at level of umbilicus (separate equations derived per covariate), b1 and b2 are regression coefficients and a is the intercept for each covariate.

1. Buckley RG, Stehman CR, Dos Santos FL, Riffenburgh RH, Swenson A, Mjos N, et al. Bedside method to estimate actual body weight in the Emergency Department. J Emerg Med. 2012;42(1):100-4.

2. Lorenz MW, Graf M, Henke C, Hermans M, Ziemann U, Sitzer M, et al. Anthropometric approximation of body weight in unresponsive stroke patients. J Neurol Neurosurg Psychiatry. 2007;78(12):1331-6.

3. Chumlea WC, Guo S, Roche AF, Steinbaugh ML. Prediction of body weight for the nonambulatory elderly from anthropometry. J Am Diet Assoc. 1988;88(5):564-8.

4. Bernal-Orozco MF, Vizmanos B, Hunot C, Flores-Castro M, Leal-Mora D, Cells A, et al. Equation to estimate body weight in elderly Mexican women using anthropometric measurements. Nutr Hosp. 2010;25(4):648-55.

5. Balode A, Stolarova A, Villerusa A, Zepa D, Kalnins I, Vētra J. Estimation of body weight and stature in Latvian hospitalized seniors. Papers on Anthropology. 2015;24(2).

6. Donini LM, de Felice MR, de Bernardini L, Ferrari G, Rosano A, de Medici M, et al. Body weight estimation in the Italian elderly. J Nutr Health Aging. 1998;2(2):92-5.

7. Lin BW, Yoshida D, Quinn J, Strehlow M. A better way to estimate adult patients' weights. Am J Emerg Med. 2009;27(9):1060-4.

8. Atiea JA, Haboubi NY, Hudson PR, Sastry BD. Body weight estimation of elderly patients by nomogram. J Am Geriatr Soc. 1994;42(7):763-5.

9. Cattermole GN, Graham CA, Rainer TH. Mid-arm circumference can be used to estimate weight of adult and adolescent patients. Emerg Med J. 2017;34(4):231-6.

10. Jung MY, Chan MS, Chow VS, Chan YT, Leung PF, Leung EM, et al. Estimating geriatric patient's body weight using the knee height caliper and mid-arm circumference in Hong Kong Chinese. Asia Pac J Clin Nutr. 2004;13(3):261-4.

11. Rabito EI, Mialich MS, Martinez EZ, Garcia RW, Jordao AA, Jr., Marchini JS. Validation of predictive equations for weight and height using a metric tape. Nutr Hosp. 2008;23(6):614-8.
