# Supplementary material for: Estimation of body weight using anthropometric parameters in Sri Lankan hospitalized adult patients
Source: PLoS One. 2023 Sep 1;18(9):e0290895. doi: 10.1371/journal.pone.0290895 (PMC10473512; doi:10.1371/journal.pone.0290895)
Supplement: S2 Table — (DOCX) [file pone.0290895.s004.docx]

Supplementary Table 2. Definitions of anthropometric measurements

| Parameter | Measurement | Reference article |
| --- | --- | --- |
| Mid arm circumference | Level at midpoint between tip of acromioclavicular eminent to tip of olecranon of elbow of nondominant arm | (1-6) |
| Neck circumference | Level at cricoid cartilage in anterior and midpoint between external occipital protuberance and tip of spinous process of 7th cervical spine (vertebral prominence at root of neck) in posterior | (4, 7) |
| Chest circumference | Circumference of chest just beneath the mammary or pectoral fold during expiration | (7) |
| Abdominal circumference | Circumference of abdomen at the level of the umbilicus | (3, 4, 7, 8) |
| Waist circumference | Narrowest part of abdominal circumference between the lower part of the rib cage and the supra-iliac crest | (9) |
| Hip circumference | Widest part of hip, level of pubic symphysis at anterior and ischial tuberosity at posterior | (3, 4, 8) |
| Thigh circumference | Level at midpoint between inguinal point and upper border of patella | (4) |
| Calf circumference | Level at midpoint between heel and upper most point of femur condyles (approximately 4 cm proximal to the patella) | (1-4) |
| triceps skinfold thickness | Outer aspect of the arm, midway from the tip of the acromial process to the olecranon | (1, 2, 10) |
| Subscapular skinfold thickness | Below the tip of the scapula | (1, 2, 10) |
| Waist skinfold thickness | Measured just to the left of the umbilicus | (10) |
| Knee height | Proximally by the thigh prominence with the knee bent at 90° and distally by the plantar aspect of the foot at the heel with the ankle bent at 90° | (5, 6) |
| Tibial length | From the centre of the medial malleolus to the tibial tuberosity | (1, 2, 7) |

1. Chumlea WC, Guo S, Roche AF, Steinbaugh ML. Prediction of body weight for the nonambulatory elderly from anthropometry. J Am Diet Assoc. 1988;88(5):564-8.

2. Bernal-Orozco MF, Vizmanos B, Hunot C, Flores-Castro M, Leal-Mora D, Cells A, et al. Equation to estimate body weight in elderly Mexican women using anthropometric measurements. Nutr Hosp. 2010;25(4):648-55.

3. Balode A, Stolarova A, Villerusa A, Zepa D, Kalnins I, Vētra J. Estimation of body weight and stature in Latvian hospitalized seniors. Papers on Anthropology. 2015;24(2).

4. Chittawatanarat K, Pruenglampoo S, Trakulhoon V, Ungpinitpong W, Patumanond J. Development of gender- and age group-specific equations for estimating body weight from anthropometric measurement in Thai adults. Int J Gen Med. 2012;5:65-80.

5. Lin BW, Yoshida D, Quinn J, Strehlow M. A better way to estimate adult patients' weights. Am J Emerg Med. 2009;27(9):1060-4.

6. Jung MY, Chan MS, Chow VS, Chan YT, Leung PF, Leung EM, et al. Estimating geriatric patient's body weight using the knee height caliper and mid-arm circumference in Hong Kong Chinese. Asia Pac J Clin Nutr. 2004;13(3):261-4.

7. Buckley RG, Stehman CR, Dos Santos FL, Riffenburgh RH, Swenson A, Mjos N, et al. Bedside method to estimate actual body weight in the Emergency Department. J Emerg Med. 2012;42(1):100-4.

8. Lorenz MW, Graf M, Henke C, Hermans M, Ziemann U, Sitzer M, et al. Anthropometric approximation of body weight in unresponsive stroke patients. J Neurol Neurosurg Psychiatry. 2007;78(12):1331-6.

9. Lohman TJ, Roache A, Martorell R, editors. Anthropometric Standardization Reference Manual1988.

10. Atiea JA, Haboubi NY, Hudson PR, Sastry BD. Body weight estimation of elderly patients by nomogram. J Am Geriatr Soc. 1994;42(7):763-5.
