# Supplementary material for: Estimation of body weight using anthropometric parameters in Sri Lankan hospitalized adult patients
Source: PLoS One. 2023 Sep 1;18(9):e0290895. doi: 10.1371/journal.pone.0290895 (PMC10473512; doi:10.1371/journal.pone.0290895)
Supplement: S3 Table — (DOCX) [file pone.0290895.s005.docx]

Supplementary Table 3. Anthropometric measurements of all patients in the derivation cohort (n = 502)

| **Males** | | | |
| --- | --- | --- | --- |
|  | Mean | Std. Deviation | N |
| Actual weight | 61.19 | 12.856 | 249 |
| Mid arm circumference | 27.215 | 3.8801 | 249 |
| Neck circumference | 36.677 | 3.0935 | 249 |
| Chest circumference | 87.01 | 9.018 | 249 |
| Abdominal circumference | 82.771 | 12.4028 | 249 |
| Waist circumference | 78.335 | 10.9938 | 249 |
| Hip circumference | 82.13 | 9.598 | 249 |
| Thigh circumference | 40.944 | 5.4931 | 249 |
| Calf circumference | 32.614 | 3.4644 | 249 |
| Knee height | 50.729 | 2.6402 | 249 |
| Tibial length | 36.480 | 2.6647 | 249 |
| Triceps skin fold thickness | 12.97 | 4.701 | 249 |
| Subscapular skin fold thickness | 16.78 | 5.933 | 249 |
| Waist skinfold thickness | 19.43 | 6.996 | 249 |
|  | | | |
| **Females** | | | |
|  | Mean | Std. Deviation | N |
| Actual weight | 57.85 | 11.794 | 253 |
| Mid arm circumference | 28.067 | 4.7875 | 253 |
| Neck circumference | 34.573 | 3.7575 | 253 |
| Chest circumference | 97.29 | 12.938 | 253 |
| Abdominal circumference | 97.192 | 14.2022 | 253 |
| Waist circumference | 91.138 | 13.5052 | 253 |
| Hip circumference | 105.38 | 14.539 | 253 |
| Thigh circumference | 41.340 | 6.0817 | 253 |
| Calf circumference | 33.014 | 4.4571 | 253 |
| Knee height | 41.688 | 2.7316 | 253 |
| Tibial length | 33.368 | 2.8388 | 253 |
| Triceps skin fold thickness | 16.15 | 7.301 | 253 |
| Subscapular skin fold thickness | 16.71 | 8.435 | 253 |
| Waist skinfold thickness | 14.85 | 6.662 | 253 |
|  | | | |
