# Supplementary material for: Estimation of body weight using anthropometric parameters in Sri Lankan hospitalized adult patients
Source: PLoS One. 2023 Sep 1;18(9):e0290895. doi: 10.1371/journal.pone.0290895 (PMC10473512; doi:10.1371/journal.pone.0290895)
Supplement: S4 Table — (DOCX) [file pone.0290895.s006.docx]

Supplementary Table 4. Percentage errors between actual body weight and the estimated body weight calculated from each of the equations listed in Table 1 (Development cohort, n=502).

| Equation | ≥ 5% error | | ≥10% error | | ≥20% error | |
| --- | --- | --- | --- | --- | --- | --- |
|  | Instances | % | Instances | % | Instances | % |
| Buckley et al. (1) | 318 | 63.35 | 177 | 35.26 | 43 | 8.57 |
| Chumlea et al. (2) | 344 | 68.53 | 196 | 39.04 | 51 | 10.16 |
| Chumlea et al. (2) | 330 | 65.74 | 199 | 39.64 | 41 | 8.17 |
| Chumlea et al. (2) | 358 | 71.31 | 245 | 48.80 | 93 | 18.53 |
| Bernal – Orozco et al. (3)* | 209 | 82.61 | 165 | 65.22 | 79 | 31.23 |
| Balode et al. (4) | 383 | 76.29 | 278 | 55.38 | 129 | 25.70 |
| Donini et al. (5) | 407 | 81.08 | 309 | 61.55 | 158 | 31.47 |
| Lin et al. (6) | 350 | 69.72 | 223 | 44.42 | 80 | 15.94 |
| Atiea et al. (7) | 354 | 70.52 | 213 | 42.43 | 73 | 14.54 |
| Cattermole et al. (8) | 395 | 78.69 | 277 | 55.18 | 131 | 26.10 |
| Jung et al. (9) | 369 | 73.51 | 261 | 51.99 | 102 | 20.32 |
| Rabito et al. (10) | 360 | 71.71 | 241 | 48.01 | 76 | 15.14 |

*Used for females only (n= 253)

1. Buckley RG, Stehman CR, Dos Santos FL, Riffenburgh RH, Swenson A, Mjos N, et al. Bedside method to estimate actual body weight in the Emergency Department. J Emerg Med. 2012;42(1):100-4.

2. Chumlea WC, Guo S, Roche AF, Steinbaugh ML. Prediction of body weight for the nonambulatory elderly from anthropometry. J Am Diet Assoc. 1988;88(5):564-8.

3. Bernal-Orozco MF, Vizmanos B, Hunot C, Flores-Castro M, Leal-Mora D, Cells A, et al. Equation to estimate body weight in elderly Mexican women using anthropometric measurements. Nutr Hosp. 2010;25(4):648-55.

4. Balode A, Stolarova A, Villerusa A, Zepa D, Kalnins I, Vētra J. Estimation of body weight and stature in Latvian hospitalized seniors. Papers on Anthropology. 2015;24(2).

5. Donini LM, de Felice MR, de Bernardini L, Ferrari G, Rosano A, de Medici M, et al. Body weight estimation in the Italian elderly. J Nutr Health Aging. 1998;2(2):92-5.

6. Lin BW, Yoshida D, Quinn J, Strehlow M. A better way to estimate adult patients' weights. Am J Emerg Med. 2009;27(9):1060-4.

7. Atiea JA, Haboubi NY, Hudson PR, Sastry BD. Body weight estimation of elderly patients by nomogram. J Am Geriatr Soc. 1994;42(7):763-5.

8. Cattermole GN, Graham CA, Rainer TH. Mid-arm circumference can be used to estimate weight of adult and adolescent patients. Emerg Med J. 2017;34(4):231-6.

9. Jung MY, Chan MS, Chow VS, Chan YT, Leung PF, Leung EM, et al. Estimating geriatric patient's body weight using the knee height caliper and mid-arm circumference in Hong Kong Chinese. Asia Pac J Clin Nutr. 2004;13(3):261-4.

10. Rabito EI, Mialich MS, Martinez EZ, Garcia RW, Jordao AA, Jr., Marchini JS. Validation of predictive equations for weight and height using a metric tape. Nutr Hosp. 2008;23(6):614-8.
