# Supplementary material for: Estimation of body weight using anthropometric parameters in Sri Lankan hospitalized adult patients
Source: PLoS One. 2023 Sep 1;18(9):e0290895. doi: 10.1371/journal.pone.0290895 (PMC10473512; doi:10.1371/journal.pone.0290895)
Supplement: S5 Table — (DOCX) [file pone.0290895.s007.docx]

Supplementary Table 5. The correlation of each anthropometric measurement with actual body weight according to different patterns of distributions.

| Anthropometric measurement | R^2^ of line of best fit according to each distribution pattern | | | |
| --- | --- | --- | --- | --- |
|  | Linear | Logarithmic | Quadratic | Exponential |
| Mid arm circumference | 0.588 | 0.585 | 0.589 | 0.589 |
| Neck circumference | 0.472 | 0.448 | 0.492 | 0.473 |
| Chest circumference | 0.449 | 0.455 | 0.455 | 0.456 |
| Abdominal circumference | 0.378 | 0.376 | 0.378 | 0.376 |
| Waist circumference | 0.384 | 0.388 | 0.388 | 0.387 |
| Hip circumference | 0.203 | 0.206 | 0.204 | 0.204 |
| Thigh circumference | 0.556 | 0.548 | 0.556 | 0.553 |
| Calf circumference | 0.532 | 0.522 | 0.532 | 0.53 |
| triceps skinfold thickness | 0.278 | 0.293 | 0.297 | 0.276 |
| Subscapular skinfold thickness | 0.397 | 0.365 | 0.403 | 0.392 |
| Waist skinfold thickness | 0.41 | 0.351 | 0.418 | 0.408 |
| Knee height | 0.066 | 0.064 | 0.069 | 0.066 |
| Tibial length | 0.101 | 0.099 | 0.102 | 0.098 |
