# Supplementary material for: Estimation of body weight using anthropometric parameters in Sri Lankan hospitalized adult patients
Source: PLoS One. 2023 Sep 1;18(9):e0290895. doi: 10.1371/journal.pone.0290895 (PMC10473512; doi:10.1371/journal.pone.0290895)
Supplement: S6 Table — a. Prediction of body weight with selected anthropometric measurements–summary of linear regression for males. b. Prediction of body weight with selected anthropometric measurements–summary of linear regression for females. (DOCX) [file pone.0290895.s008.docx]

Supplementary Table 6 a. Prediction of body weight with selected anthropometric measurements – summary of linear regression for males

| **Model Summary^a,c^** | | | | | | | | | |
| --- | --- | --- | --- | --- | --- | --- | --- | --- | --- |
| Model | R | R Square | Adjusted R Square | Std. Error of the Estimate | Change Statistics | | | | |
|  |  |  |  |  | R Square Change | F Change | df1 | df2 | Sig. F Change |
| 1 | .908^b^ | .824 | .822 | 5.430 | .824 | 286.570 | 4 | 244 | .000 |
| a. Gender = Male | | | | | | | | | |
| b. Predictors: (Constant), Triceps skin fold thickness, Tibial length, Abdominal circumference, Mid arm circumference | | | | | | | | | |
| c. Dependent Variable: Actual Weight | | | | | | | | | |

| **Coefficients^a,b^** | | | | | | | | | |
| --- | --- | --- | --- | --- | --- | --- | --- | --- | --- |
| Model | | Unstandardized Coefficients | | Standardized Coefficients | t | Sig. | Correlations | | |
|  |  | B | Std. Error | Beta |  |  | Zero-order | Partial | Part |
| 1 | (Constant) | -38.213 | 5.323 |  | -7.179 | .000 |  |  |  |
|  | Mid arm circumference | 1.218 | .138 | .368 | 8.831 | .000 | .818 | .492 | .237 |
|  | Abdominal circumference | .490 | .040 | .473 | 12.368 | .000 | .823 | .621 | .332 |
|  | Tibial length | .565 | .137 | .117 | 4.137 | .000 | .316 | .256 | .111 |
|  | Triceps skin fold thickness | .391 | .100 | .143 | 3.911 | .000 | .682 | .243 | .105 |
| a. Gender = Male | | | | | | | | | |
| b. Dependent Variable: Actual Weight | | | | | | | | | |

Supplementary Table 6 b. Prediction of body weight with selected anthropometric measurements – summary of linear regression for females

| **Model Summary^a,c^** | | | | | | | | | |
| --- | --- | --- | --- | --- | --- | --- | --- | --- | --- |
| Model | R | R Square | Adjusted R Square | Std. Error of the Estimate | Change Statistics | | | | |
|  |  |  |  |  | R Square Change | F Change | df1 | df2 | Sig. F Change |
| 1 | .865^b^ | .747 | .741 | 5.999 | .747 | 121.306 | 6 | 246 | .000 |
| a. Gender = Female | | | | | | | | | |
| b. Predictors: (Constant), Waist skinfold thickness, Neck circumference, Subscapular skin fold thickness, Triceps skin fold thickness, Chest circumference, Mid arm circumference | | | | | | | | | |
| c. Dependent Variable: Actual Weight | | | | | | | | | |

| **Coefficients^a,b^** | | | | | | | | | |
| --- | --- | --- | --- | --- | --- | --- | --- | --- | --- |
| Model | | Unstandardized Coefficients | | Standardized Coefficients | t | Sig. | Correlations | | |
|  |  | B | Std. Error | Beta |  |  | Zero-order | Partial | Part |
| 1 | (Constant) | -20.128 | 3.851 |  | -5.227 | .000 |  |  |  |
|  | Mid arm circumference | .968 | .137 | .393 | 7.053 | .000 | .783 | .410 | .226 |
|  | Triceps skin fold thickness | .047 | .077 | .029 | .611 | .542 | .552 | .039 | .020 |
|  | Neck circumference | .273 | .136 | .087 | 2.004 | .046 | .627 | .127 | .064 |
|  | Chest circumference | .407 | .045 | .447 | 9.040 | .000 | .795 | .499 | .290 |
|  | Subscapular skin fold thickness | -.020 | .061 | -.015 | -.334 | .739 | .547 | -.021 | -.011 |
|  | Waist skinfold thickness | .087 | .068 | .049 | 1.281 | .201 | .439 | .081 | .041 |
| a. Gender = Female | | | | | | | | | |
| b. Dependent Variable: Actual Weight | | | | | | | | | |
