# Supplementary material for: Estimation of body weight using anthropometric parameters in Sri Lankan hospitalized adult patients
Source: PLoS One. 2023 Sep 1;18(9):e0290895. doi: 10.1371/journal.pone.0290895 (PMC10473512; doi:10.1371/journal.pone.0290895)

Supplementary Table 7. Model diagnosis of linear regression presented in supplementary table 6 for males and females.

Linear regression in males

|  | | | | |
| --- | --- | --- | --- | --- |
|  | N | Minimum | Maximum | Mean |
| Standardized Residual | 249 | -2.62964 | 2.76056 | .0000000 |
| Standardized DFBETA Intercept | 249 | -.48781 | .25346 | -.0000359 |
| Standardized DFBETA Midarmcircumference | 249 | -.47107 | .26936 | -.0001201 |
| Standardized DFBETA Abdcircumference | 249 | -.26801 | .30639 | .0002475 |
| Standardized DFBETA Tibiallength | 249 | -.23163 | .33059 | -.0000312 |
| Standardized DFBETA Tricepskinfoldthickness | 249 | -.27212 | .27338 | -.0000191 |
| Valid N (listwise) | 249 |  |  |  |
| a. Gender = 1 | | | | |

| **Coefficients^a,b^** | | | | | | | | |
| --- | --- | --- | --- | --- | --- | --- | --- | --- |
| Model | | Unstandardized Coefficients | | Standardized Coefficients | t | Sig. | Collinearity Statistics | |
|  |  | B | Std. Error | Beta |  |  | Tolerance | VIF |
| 1 | (Constant) | -38.213 | 5.323 |  | -7.179 | <.001 |  |  |
|  | Mid arm circumference | 1.218 | .138 | .368 | 8.831 | <.001 | .415 | 2.409 |
|  | Abd circumference | .490 | .040 | .473 | 12.368 | <.001 | .492 | 2.033 |
|  | Tibial length | .565 | .137 | .117 | 4.137 | <.001 | .898 | 1.114 |
|  | Tricep skin fold thickness | .391 | .100 | .143 | 3.911 | <.001 | .538 | 1.857 |
| a. Gender = 1 | | | | | | | | |
| b. Dependent Variable: Actual Weight | | | | | | | | |

| **Model Summary^a,c^** | | | | | | | | | | |
| --- | --- | --- | --- | --- | --- | --- | --- | --- | --- | --- |
| Model | R | R Square | Adjusted R Square | Std. Error of the Estimate | Change Statistics | | | | | Durbin-Watson |
|  |  |  |  |  | R Square Change | F Change | df1 | df2 | Sig. F Change |  |
| 1 | .908^b^ | .824 | .822 | 5.4296 | .824 | 286.570 | 4 | 244 | <.001 | 1.449 |
| a. Gender = 1 | | | | | | | | | | |
| b. Predictors: (Constant), Tricep skin fold thickness, Tibial length, Abd circumference, Mid arm circumference | | | | | | | | | | |
| c. Dependent Variable: Actual Weight | | | | | | | | | | |


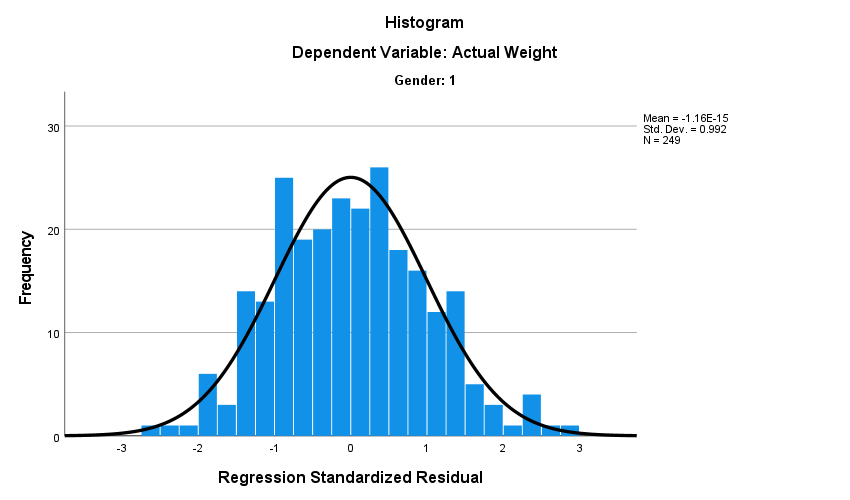


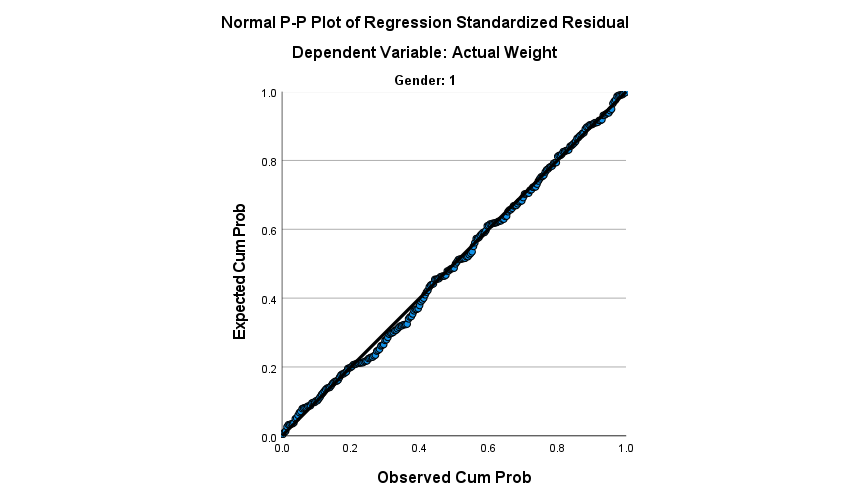


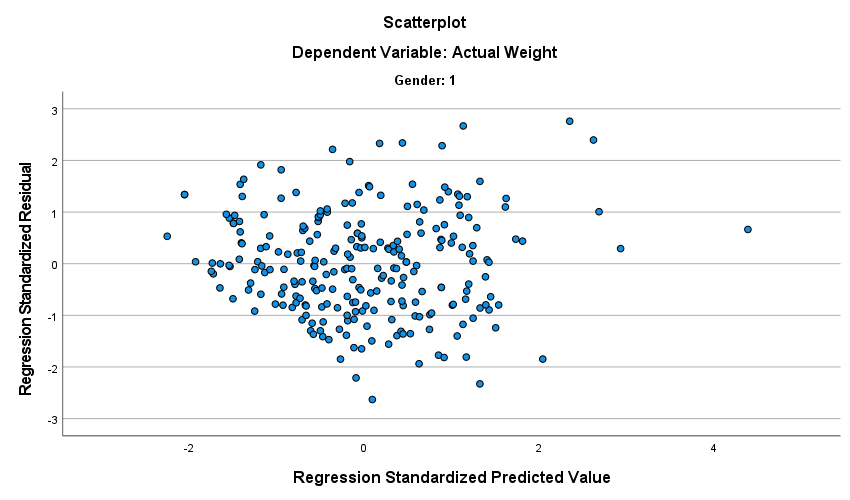


Linear regression in females

| **Descriptive Statistics^a^** | | | | |
| --- | --- | --- | --- | --- |
|  | N | Minimum | Maximum | Mean |
| Standardized Residual | 253 | -2.69953 | 3.21508 | .0000000 |
| Standardized DFBETA Intercept | 253 | -.23118 | .30459 | .0001175 |
| Standardized DFBETA Midarmcircumference | 253 | -.26527 | .35076 | .0000932 |
| Standardized DFBETA Neckcircumference | 253 | -.26270 | .30169 | -.0001170 |
| Standardized DFBETA Chestcircumference | 253 | -.41916 | .34987 | -.0000138 |
| Standardized DFBETA Subscapularskinfoldthickness | 253 | -.38859 | .34034 | .0001063 |
| Standardized DFBETA Waistskinfoldthickness | 253 | -.33109 | .41472 | -.0001368 |
| Standardized DFBETA Tricepskinfoldthickness | 253 | -.36763 | .27177 | -.0000904 |
| Valid N (listwise) | 253 |  |  |  |
| a. Gender = 2 | | | | |

| **Coefficients^a,b^** | | | | | | | | |
| --- | --- | --- | --- | --- | --- | --- | --- | --- |
| Model | | Unstandardized Coefficients | | Standardized Coefficients | t | Sig. | Collinearity Statistics | |
|  |  | B | Std. Error | Beta |  |  | Tolerance | VIF |
| 1 | (Constant) | -20.128 | 3.851 |  | -5.227 | <.001 |  |  |
|  | Mid arm circumference | .968 | .137 | .393 | 7.053 | <.001 | .331 | 3.025 |
|  | Neck circumference | .273 | .136 | .087 | 2.004 | .046 | .544 | 1.838 |
|  | Chest circumference | .407 | .045 | .447 | 9.040 | <.001 | .420 | 2.381 |
|  | Subscapular skin fold thickness | -.020 | .061 | -.015 | -.334 | .739 | .532 | 1.880 |
|  | Waist skinfold thickness | .087 | .068 | .049 | 1.281 | .201 | .700 | 1.428 |
|  | Tricep skin fold thickness | .047 | .077 | .029 | .611 | .542 | .447 | 2.238 |
| a. Gender = 2 | | | | | | | | |
| b. Dependent Variable: Actual Weight | | | | | | | | |

| **Model Summary^a,c^** | | | | | | | | | | |
| --- | --- | --- | --- | --- | --- | --- | --- | --- | --- | --- |
| Model | R | R Square | Adjusted R Square | Std. Error of the Estimate | Change Statistics | | | | | Durbin-Watson |
|  |  |  |  |  | R Square Change | F Change | df1 | df2 | Sig. F Change |  |
| 1 | .865^b^ | .747 | .741 | 5.9994 | .747 | 121.306 | 6 | 246 | <.001 | 1.418 |
| a. Gender = 2 | | | | | | | | | | |
| b. Predictors: (Constant), Triceps skin fold thickness, Neck circumference, Waist skinfold thickness, Subscapular skin fold thickness, Chest circumference, Mid arm circumference | | | | | | | | | | |
| c. Dependent Variable: Actual Weight | | | | | | | | | | |


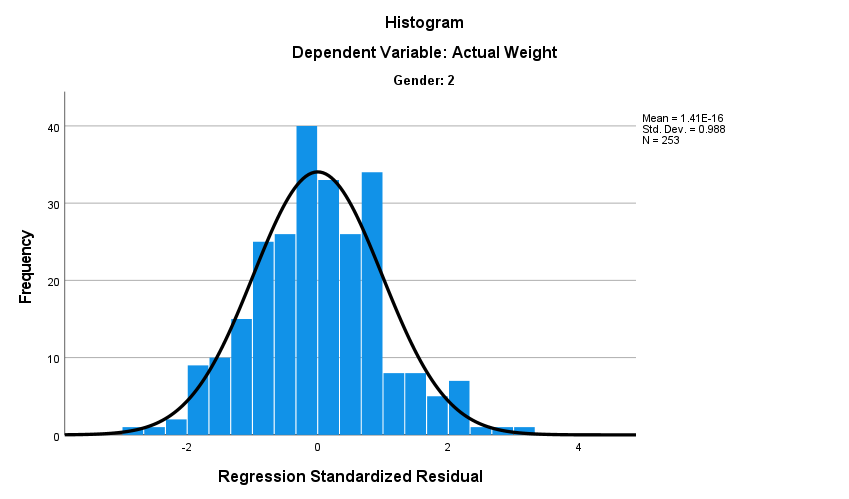


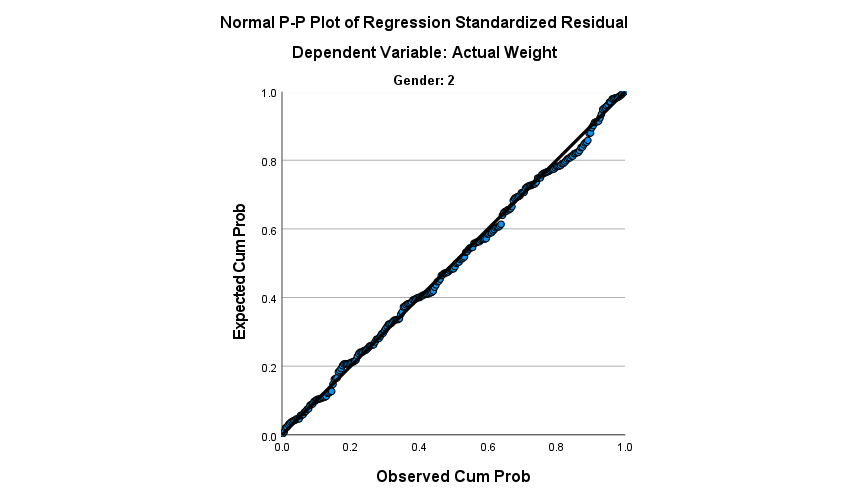


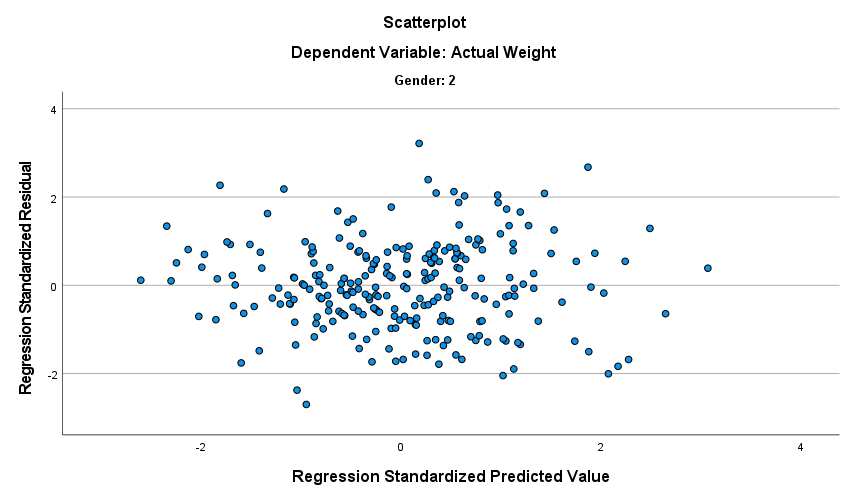

Supplement: S7 Table — (DOCX) [file pone.0290895.s009.docx]
