# Supplementary material for: Estimation of body weight using anthropometric parameters in Sri Lankan hospitalized adult patients
Source: PLoS One. 2023 Sep 1;18(9):e0290895. doi: 10.1371/journal.pone.0290895 (PMC10473512; doi:10.1371/journal.pone.0290895)
Supplement: S8 Table — (DOCX) [file pone.0290895.s010.docx]

Supplementary Table 6. Differences between validation and derivation cohorts for age, actual body weight and anthropometric measurements of interest that were selected for the regression equation

1. Males

|  | | | | | |
| --- | --- | --- | --- | --- | --- |
|  | Cohort | N | Mean | Std. Deviation | Std. Error Mean |
| Age | Development | 249 | 47.26 | 16.438 | 1.042 |
|  | Validation | 108 | 46.12 | 15.154 | 1.458 |
| Actual Weight | Development | 249 | 61.186 | 12.8556 | .8147 |
|  | Validation | 108 | 62.382 | 11.8135 | 1.1368 |
| Mid arm circumference | Development | 249 | 27.215 | 3.8801 | .2459 |
|  | Validation | 108 | 27.329 | 3.7809 | .3638 |
| Abdominal circumference | Development | 249 | 82.771 | 12.4028 | .7860 |
|  | Validation | 108 | 84.204 | 12.1797 | 1.1720 |
| Triceps skin fold thickness | Development | 249 | 12.97 | 4.701 | .298 |
|  | Validation | 108 | 12.83 | 4.211 | .405 |
| Tibial length | Development | 249 | 36.480 | 2.6647 | .1689 |
|  | Validation | 108 | 36.051 | 2.8110 | .2705 |

p>0.05 for all variables (no difference in development and validation cohorts)

1. Females

|  | | | | | |
| --- | --- | --- | --- | --- | --- |
|  | Cohort | N | Mean | Std. Deviation | Std. Error Mean |
| Age* | Development | 253 | 53.13 | 33.852 | 2.128 |
|  | Validation | 109 | 44.09 | 16.217 | 1.553 |
| Actual Weight | Development | 253 | 57.853 | 11.7937 | .7415 |
|  | Validation | 109 | 56.606 | 13.2529 | 1.2694 |
| Mid arm circumference | Development | 253 | 28.07 | 4.787 | .301 |
|  | Validation | 109 | 27.22 | 5.032 | .482 |
| Neck circumference* | Development | 253 | 34.573 | 3.7575 | .2362 |
|  | Validation | 109 | 32.977 | 3.0523 | .2924 |
| Chest circumference | Development | 253 | 97.29 | 12.938 | .813 |
|  | Validation | 109 | 95.32 | 12.986 | 1.244 |

*p<0.05 with a statistically significant difference
